# An insect brain organizes numbers on a left-to-right mental number line

**DOI:** 10.1073/pnas.2203584119

**Published:** 2022-10-17

**Authors:** Martin Giurfa, Claire Marcout, Peter Hilpert, Catherine Thevenot, Rosa Rugani

**Affiliations:** ^a^Centre de Recherches sur la Cognition Animale, Centre de Biologie Intégrative, University of Toulouse, CNRS, University Paul Sabatier, Toulouse cedex 9, 31062 France;; ^b^Institut Universitaire de France, 75231 Paris cedex 05, France;; ^c^Institute of Psychology, University of Lausanne, CH-1015 Lausanne, Switzerland;; ^d^Department of General Psychology, University of Padova, 35100 Padova, Italy

**Keywords:** numerosity, numeric representation, mental number line, honey bees, *Apis mellifera*

## Abstract

The “mental number line” (MNL) is a form of spatial numeric representation that associates small and large numbers with the left and right spaces, respectively. This spatio-numeric organization can be found in adult humans and has been related to cultural factors such as writing and reading habits. Yet, both human newborns and birds order numbers consistently with an MNL, thus raising the question of whether culture is a main explanation for MNL. Here, we explored the numeric sense of honey bees and show that after being trained to associate numbers with a sucrose reward, they order numbers not previously experienced from left to right according to their magnitude. Importantly, the location of a number on that scale varies with the reference number previously trained and does not depend on low-level cues present on numeric stimuli. We provide a series of neural explanations for this effect based on the extensive knowledge accumulated on the neural underpinnings of visual processing in honey bees and conclude that the MNL is a form of numeric representation that is evolutionarily conserved across nervous systems endowed with a sense of number, irrespective of their neural complexity.

## Significance

The ability to judge numbers exists in various vertebrate species but also in honey bees, thus raising the question of the phylogenetic origins of numerosity systems. Here, we studied if bees, like humans, organize numbers spatially from left to right according to their magnitude. As the cultural vs. biological origins of this mental number line (MNL) are a subject of debate, our study provides an important perspective for this discussion. We show that bees order numbers from left to right according to their magnitude and that the location of a number on that line varies with the reference number previously trained. Thus, the MNL is a biological numeric representation that is common to the nervous system with distant evolutionary origins.

Traditionally, the “mental number line” (MNL), which leads us to associate small numbers with the left space and large numbers with the right space (1, 2), has been considered a cultural result, mainly attributed to writing and reading habits. For instance, Iranian participants, who use an adapted form of Arabic script written and read from right to left, do not exhibit the association of small and large numbers with left and right, respectively. Yet, after spending several years in a Western culture, the MNL effect emerges in the same participants (2). Recent evidence indicates, however, that both human newborns (3, 4) and some nonhuman vertebrates (5–10) order numbers according to an MNL, thus suggesting an inborn component in this spatial number representation, an idea that is still controversial (11, 12).

Evidence from avians (5, 6, 9) indicates that the association between numbers and space is possible for brains lacking the six-layered neo-cortex, a structure considered an essential prerequisite for higher-order cognition. Yet, whether this spatial sorting of numbers is restricted to vertebrates or exists also in the brain of invertebrates despite its considerable reduction in size and number of neurons remains to be determined. Honey bees represent an attractive species to address this question because their numeric sense (13–18) has attracted wide attention due to its similarities with that of some vertebrates (19). Although they have the capacity to process separately numeric magnitudes and space (19, 20), whether their numeric sense organizes numbers in the form of an MNL remains unknown. Here, we investigated this question and show that honey bees trained to associate numbers with a sucrose reward order numbers not previously experienced from left to right according to their magnitude and that the location of a number on that scale depends on the reference number previously trained.

## Results

**Fig. 1.**
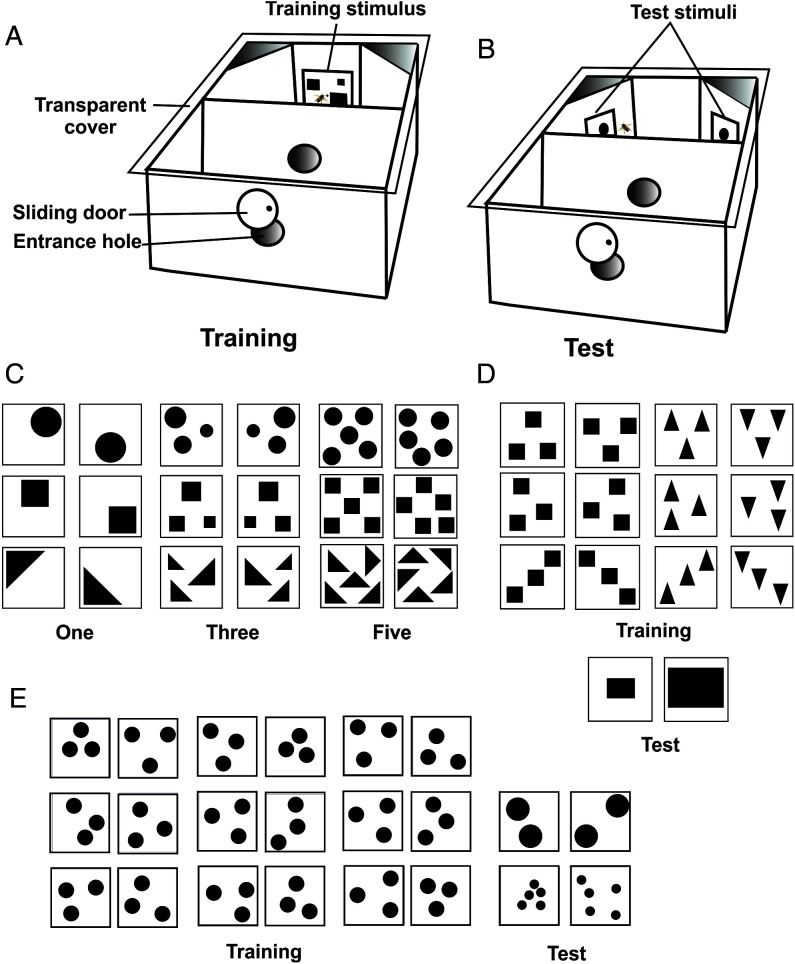
(*A*) Overall view of the experimental setup during training. The wooden box was covered by a transparent cover, which allowed observing the behavior of the bees accessing the inner compartments. After passing the first inner compartment, the focal bee faced a training stimulus placed in the middle of the back wall. An Eppendorf tip delivered sucrose solution in the middle of the image. (*B*) Overall view of the experimental setup during a test. After passing the first inner compartment, the focal bee faced two lateral walls displaying the same test alternative on each side. The test stimuli were novel to the trained bee, i.e. they were never experienced during the training. No reward was provided during the test. The first choice and the cumulative choices performed during 40 s were recorded. (*C*) Examples of stimuli used in the first and second experiment. (*D*) Stimuli used in the third experiment. (*E*) Examples of stimuli used in the fourth experiment.

In all experiments, the same general methods were used. Briefly, individually marked, free-flying honey bees were trained to fly into a wooden box to collect sucrose solution on a visual target with controlled properties. Bees had to fly into a first compartment through an entrance hole in the box and then into a second compartment through a second hole in the middle of an internal wall (Fig. 1*A*). The target, centered on the back wall of the second compartment, was a white square displaying a fixed number of items differing in shape (triangles, squares, circles; Fig. 1*C*). Bees were trained over 30 consecutive visits to associate a specific number of items with a sucrose reward. The trained number remained constant, but the shape of elements displayed varied pseudo randomly; at the end of training, circles, squares, and triangles appeared 10 times each in variable spatial arrays. Sucrose solution was delivered by an Eppendorf tip inserted in the middle of the target. After completing the 30 visits, the central target was removed and two identical targets, displaying the same number and the same shape, were presented simultaneously, one on the left and the other on the right of the back wall (5) (Fig. 1*B*). They were flipped such that the stimulus on the right provided a mirror image of the stimulus on the left. The bee was then subjected to a dual-choice extinction test (i.e., no reward provided). The first choice as well as the cumulative choices performed during 40 s were recorded during each test. Overall, eight experiments were conducted involving a total of 134 bees and 298 observations, given that several tests were repeated. Three of these experiments were conceived as controls in which bees were trained and tested with the same number to verify that uncontrolled cues did not induce side biases (i.e., left vs. right). The other four experiments addressed the question of whether bees order numbers according to an MLN representation.

In the three control experiments, independent groups of 12 bees were trained during 30 consecutive visits (i.e., 30 learning trials) on One, Three or Five (Fig. 1 *A* and *C* and *SI Appendix*, Fig. 1) and then tested with the same number as they were trained on. The training stimulus was a central target image displaying the number trained (Fig. 1*A*). When training stimuli had more than one item (i.e., three or five), each item within a target had a different surface area but targets displaying the same number presented the same cumulative area. Training stimuli changed from visit to visit (i.e., squares, circles, triangles, balanced along training). Test stimuli were identical, nonrewarded, mirrored images displayed twice on the lateral panels (Fig. 1*B*). In these control experiments, no significant preference for left or right was expected. Our results showed that this was indeed the case (Tables 1 and 2; training One/test One first choice: empirical proportion of left choices, 0.58, *P* = 0.541, 95% CI, 0.37 to 0.78; cumulative choices: regression coefficient from a linear mixed model (defined in *Materials and Methods*), γ_1_ = 0.1, *P* = 0.858, conditional *R*^2^ [*R*^2^c] < 0.1%. For training Three/test Three, first choice: empirical proportion of left choices, 0.54, *P* = 0.839, 95% CI, 0.33 to 0.74; cumulative choices: γ_1_ = 0.2, *P* = 0.672, *R*^2^c < 0.1%. For training Five/test Five, first choice: empirical proportion of left choices, 0.50, *P* = 0.999, 95% CI, 0.29 to 0.71; cumulative choices: γ_1_ = 0.4, *P* = 0.267, *R*^2^c < 0.1%). These results show that training did not induce side biases per se.

**Table 1.** Results of the exact binomial tests used to assess the deviance of the bees’ first choice from a random distribution between left and right sides

	First choices						
	Train on	Test on	Count L/R	Empirical proportion*	Expected proportion	*P*	95% CI
Control experiments	One	One	14/10	0.58	0.50	0.541	0.37 to 0.78
	Three	Three	13/11	0.54	0.50	0.839	0.33 to 0.74
	Five	Five	12/12	0.50	0.50	1.000	0.29 to 0.71
Experiment 1	Three	One	26/2	**0.93**	0.50	<0.001	0.76 to 0.99
	Three	Five	6/22	**0.79**	0.50	0.004	0.59 to 0.92
Experiment 2	One	Three	9/23	**0.72**	0.50	0.020	0.53 to 0.86
	Five	Three	22/8	**0.73**	0.50	0.016	0.54 to 0.88
Experiment 2-bis	One	Three	3/15	**0.83**	0.50	0.0080	0.59 to 0.96
	Five	Three	14/2	**0.88**	0.50	0.004	0.62 to 0.98
Experiment 3	Three	One	22/8	**0.73**	0.50	0.016	0.54 to 0.88
Experiment 4	Three	Two	16/5	**0.76**	0.50	0.027	0.52 to 0.92
	Three	Five	5/16	**0.76**	0.50	0.027	0.52 to 0.92

L, flying to the left; R, flying to the right; the empirical proportion was calculated based on the actual L/R proportion. The expected proportion is in all cases 0.50 corresponding to a random choice between alternatives. 95% CI, 95% confidence interval.

^*^Significant coefficients are in bold (*P* < 0.05; two-tailed).

**Table 2.** Results of the multilevel-model analyses used for the bees’ cumulative choices

	Cumulative choices								
	Train on	Test on	M (SD), L/R	Est*	SE	*t*	*P*	95% CI	*R*^2^c (%)
Control experiments	One	One	5.3 (1.6)/5.3 (1.6)	0.1	0.46	0.2	0.858	−0.8 to 1.0	<0.01
	Three	Three	5.0 (1.6)/5.2 (1.8)	0.2	0.49	0.4	0.672	−0.8 to 1.2	<0.01
	Five	Five	4.8 (1.3)/5.3 (1.3)	0.4	0.37	1.1	0.267	−0.3 to 1.2	<0.01
Experiment 1	Three	One	6.7 (2.2)/3.4 (1.6)	**−3.3**	0.47	−7.1	<0.001	−4.3 to −2.4	52.4
	Three	Five	4.6 (1.8)/7.5 (1.9)	**2.9**	0.47	6.1	<0.001	1.9 to 3.7	43.5
Experiment 2	One	Three	5.0 (1.8)/9.5 (2.2)	**4.5**	0.45	9.9	<0.001	3.6 to 5.4	64.5
	Five	Three	7.9 (2.6)/5.2 (1.7)	**−2.8**	0.52	−5.4	<0.001	−3.9 to −1.8	42.0
Experiment 2-bis	One	Three	4.6 (2.2)/10.8 (3.2)	**6.2**	0.64	7.1	<0.001	4.5 to 8.0	57.6
	Five	Three	11.9 (3.2)/4.8 (2.3)	**−7.1**	0.95	−7.5	<0.001	−9.0 to −5.2	65.4
Experiment 3	Three	One	5.2 (1.8)/2.2 (1.0)	**−3.0**	0.35	−8.5	<0.001	−3.7 to −2.3	58.3
									**Adj *R*^2^ (%)**
Experiment 4	3	2	4.9 (1.0)/2.1 (0.8)	**−2.8**	0.28	−9.9	<0.001	−3.3 to −2.2	70.5
	3	5	2.6 (1.5)/4.6 (1.5)	**2.0**	0.47	4.3	<0.001	1.1 to 3.0	30.2

L, flying to the left; R, flying to the right; M(SD), mean(standard deviation); Est, unstandardized estimates; SE, standard error; R^2^c, conditional R^2^ (i.e., variance explained by the entire model); Adj. R^2^, adjusted R^2^ (no repeated measure was used in experiment 4).

^*^Significant coefficients are in bold (*P* < 0.05; two-tailed).

**Fig. 2.**
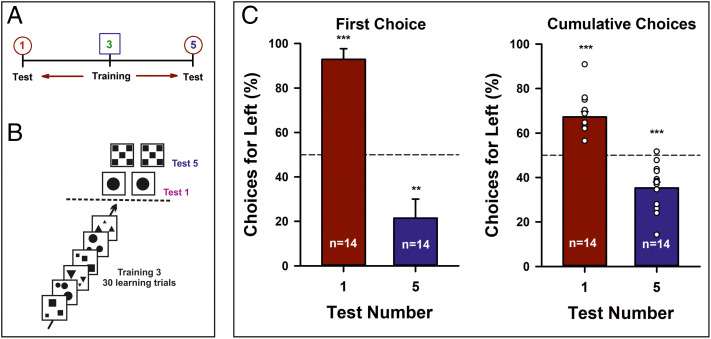
First experiment. (*A*) Experimental protocol. Bees (*n* = 14) were trained on Three and then tested on One or on Five. (*B*) Experimental schedule (simplified). Bees were trained during 30 consecutive visits to the maze with a Three numerosity rewarded. Training stimuli changed from visit to visit but the Three numerosity remained constant and was associated with a sucrose reward. Once the training finished, bees were presented with two identical test stimuli on the left and right of the decision chamber. Test stimuli were not rewarded. They were tested with a One numerosity and with a Five numerosity in separate tests, with three refreshment trials interspersed between tests. Each test was repeated twice using different shapes. (*C*) *Left*: Performance of both groups of bees when their first choice was recorded during a test opposing either One vs. One (red bar) or Five vs. Five (blue bar). The figure shows the percentage of the times a bee chose the *Left* panel. In the small-number test (One vs. One), bees trained on Three preferred the *Left* panel. In the large-number test (Five vs. Five), bees trained on Three preferred the *Right* panel. *Right*: Same as in the *Left* panel but for the cumulative choices recorded during 40 s in each test. See Statistical Analysis in *Materials and Methods*. **P = 0.004; ***P < 0.001.

We then asked if an MLN representation exists in bees trained on a given number and tested on a different number. In a first experiment, each bee (*n* = 14) was trained on Three and then tested four times in the absence of a reward, with three refreshment trials interspersed between tests. In two of the four tests, the lateral panels presented one item (Fig. 2*A*), a number smaller than the one that was trained, whereas in the other two tests, the panels displayed five items (Fig. 2*A*), a number larger than the one that was trained. Test alternatives presented the same area as the training stimuli (*SI Appendix*, Fig. 2). The shapes chosen for a given test (e.g., circle, square, or triangle) were identical mirrored images. They varied randomly between tests and bees (Fig. 2*B*). The sequence of the four tests also varied randomly from bee to bee. Significant test preferences for One on the left and for Five on the right would be consistent with a left-to-right number ordering.

When bees were tested on One after being trained to Three (Fig. 2*C*, red bars), they preferred significantly One on the left (Tables 1 and 2; training Three/test One, first choice: empirical proportion of left choices, 0.93, *P* < 0.001, 95% CI, 0.76 to 0.99; cumulative choices: γ_1_ = −3.3, *P* < 0.001, *R*^2^c = 52.4%). Yet, after the same training, they preferred Five on the right (Fig. 2*C*, blue bars; Tables 1 and 2; training Three/test Five, first choice: empirical proportion of right choices, 0.79, *P* = 0.004, 95% CI, 0.59 to 0.92; cumulative choices: γ_1_ = 2.9, *P* < 0.001, *R*^2^c = 43.5%). Thus, bees having Three as reference ordered One on the left and Five on the right.

**Fig. 3.**
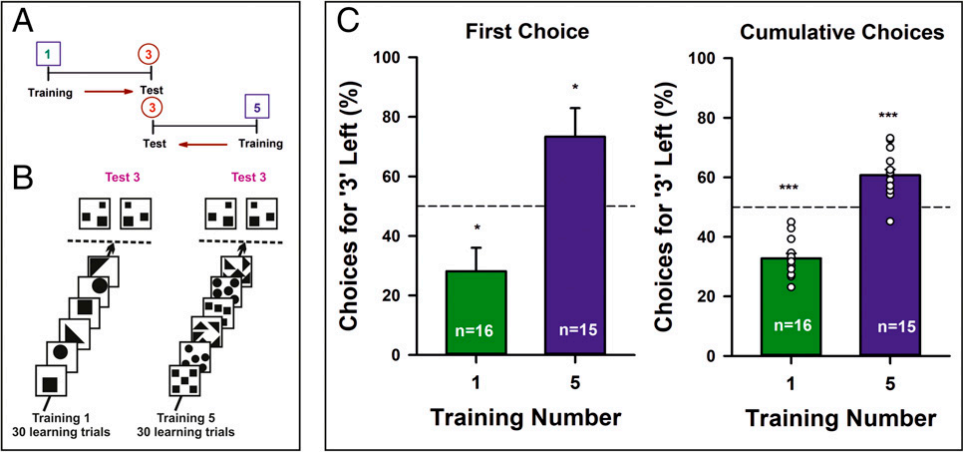
Second experiment. (*A*) Experimental protocol. Bees were either trained on One (*n* = 16) or on Five (*n* = 15) and then tested on Three. (*B*) Experimental schedule (simplified). Two groups of bees were trained during 30 consecutive visits to the maze with either a One numerosity rewarded or with a Five numerosity rewarded. Training stimuli changed from visit to visit, but the trained numerosity remained constant and was associated with a sucrose reward for each group. Once the training finished, bees were presented with two identical test stimuli displaying three items in the absence of reward. They were tested again with a Three numerosity displayed by means of different shapes (e.g., circles), with three refreshment trials interspersed between tests. (*C*) *Left*: Performance of both groups of bees when their first choice was recorded during a test opposing Three vs. Three after training to One (green bar) or to Five (blue bar). The figure shows the percentage of the times a bee chose the left panel. When the reference number was One, bees preferred the *Right* panel, whereas they preferred the *Left* panel when the reference number was Five. *Right*: Same as in the *Left* panel but for the cumulative choices recorded during 40 s in each test. See Statistical Analysis in *Materials and Methods*. *P < 0.025; ***P < 0.001.

To determine if the association of a given number with a spatial location was absolute or depended on the number used as reference, we performed a second experiment. A group of bees was trained with targets displaying one item (*n* = 16) while another group was trained with targets displaying five items (*n* = 15; Fig. 3*A*). On testing, both groups faced two panels displaying three items (Fig. 3*B*). Thus, the test alternatives were identical for both groups, but their reference number (One or Five) was different. Two tests with three interspersed refreshment trials were performed for each group, varying the item type from test to test (e.g., circle, square, or triangle). This experiment tested if the MNL is relative to the reference number experienced during the training, in which case Three on the right would be preferred after training to One, while Three on the left would be preferred after training to Five.

When bees trained on One were tested with Three, they preferred Three on the right (Tables 1 and 2; training One/test Three, first choice: empirical proportion of right choices, 0.72, *P* = 0.02, 95% CI, 0.53 to 0.86; cumulative choices: γ_1_ = 4.5, *P* < 0.001, *R*^2^c = 64.5%), whereas the second group of bees trained on Five preferred Three on the left (Tables 1 and 2; training Five/test Three, first choice: empirical proportion of left choices, 0.73, *P* = 0.016, 95% CI, 0.54 to 0.88; cumulative choices: γ_1_ = −2.8, *P* < 0.001, *R*^2^c = 42.0%). This experiment is also suitable for blind testing, given the two possible outcomes for the same Three numerosity depending on the reference number. We thus repeated the experiment with one experimenter performing the training and a second experimenter performing the tests while being blind to the training (experiment 2-bis; Tables 1 and 2). Again, bees trained on One (*n* = 9) preferred Three on the right (first choice: empirical proportion of right choices, 0.83, *P* = 0.008, 95% CI, 0.59 to 0.96; cumulative choices: γ_1_ = 6.2, *P* < 0.001, *R*^2^c < 57.6%) while bees trained on Five (*n* = 8) preferred Three on the left (first choice: empirical proportion of left choices, 0.88, *P* = 0.004, 95% CI, 0.62 to 0.98; cumulative choices: γ_1_ = −7.1, *P* < 0.001, *R*^2^c < 65.4%). These results exclude spurious cuing during the tests and demonstrate overall that the association of a number with the right or left side of space was not absolute but depended on the magnitude of the reference number.

**Fig. 4.**
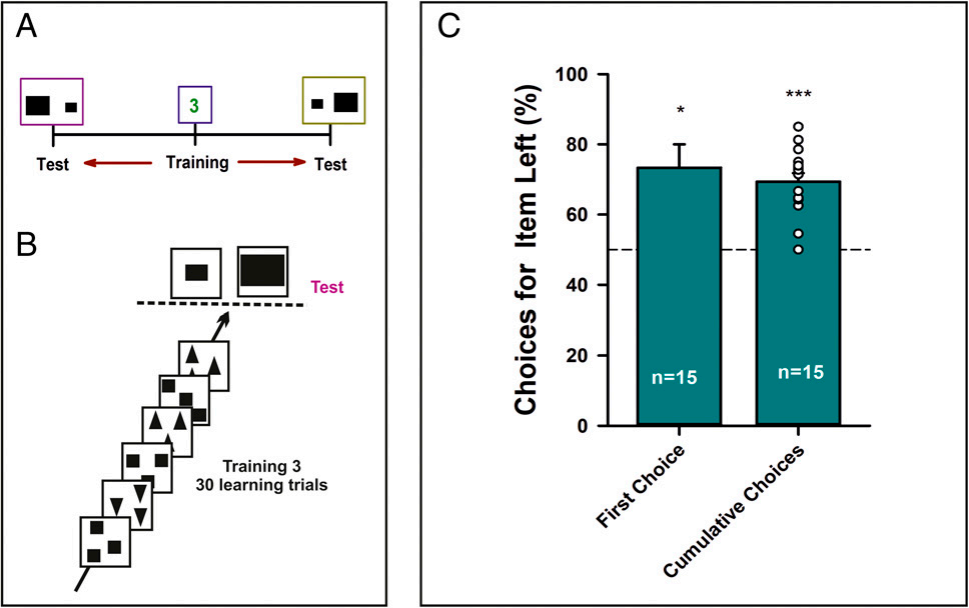
Third experiment. (*A*) Experimental protocol. Bees (*n* = 15) were trained on Three and then tested with two rectangles differing in perimeter and area. Bees had the choice between the small rectangle and the large rectangle whose perimeter matched the total perimeter of the training stimuli. (*B*) Experimental schedule (simplified). Bees were trained during 30 consecutive visits to the maze with a Three numerosity rewarded. Training stimuli changed from visit to visit but the trained numerosity remained constant and was associated with a sucrose reward. Each training stimulus was presented twice, with squares and triangles balanced along training. Once the training finished, bees were presented with the two rectangles differing in perimeter and area. Test stimuli were not rewarded. They were tested again with the same stimuli with swapped sides, with three refreshment trials interspersed between tests. (*C*) Performance of bees when their first choice was recorded during a test opposing the two rectangles or when the cumulative choices were recorded during 40 s. The figure shows the percentage of the times a bee chose the left item, be it the small or the large rectangle. Bees always preferred the *Left* panel, irrespective of the stimulus displayed. *P < 0.02; ***P < 0.001.

The previous experiments controlled for the total area of the patterns, which was the same in all images. To control for a further nonnumeric cue such as perimeter, we performed a third experiment in which bees (*n* = 15) were trained with three variable items, squares, or triangles with different spatial arrays (Fig. 1*D*, Training; *SI Appendix*, Fig. 3). Each item had a perimeter of 8 cm so that targets had a total perimeter of 24 cm. At testing, bees faced two panels, each displaying one rectangle horizontally oriented. One of the rectangles was smaller with a 12-cm perimeter, while the other had a 24-cm perimeter (Fig. 1*D*, Test; *SI Appendix*, Fig. 3), which coincided with that of the trained stimuli. Two tests with interspersed refreshment trials were performed, swapping the sides of the large and the small rectangle between tests (Fig. 4 *A* and *B*). If the bees used nonnumeric cues such as stimulus perimeter or area, they should always prefer the larger rectangle, whose total perimeter coincided with that experienced during the training. If, on the contrary, bees used numeric cues, they should always prefer the rectangle on the left, irrespective of its perimeter and area, as their training reference number was Three and a single item was presented during the test in both panels. Bees exhibited a significant preference for the rectangle on the left, irrespective of its perimeter and area (Tables 1 and 2; training Three/test One, first choice: empirical proportion of left choices, 0.73, *P* = 0.016, 95% CI, 0.54 to 0.88; cumulative choices: γ_1_ = −3.0, *P* < 0.001, *R*^2^c = 58.3%). Thus, their choice was primarily guided by numeric magnitude and not by nonnumeric cues.

**Fig. 5.**
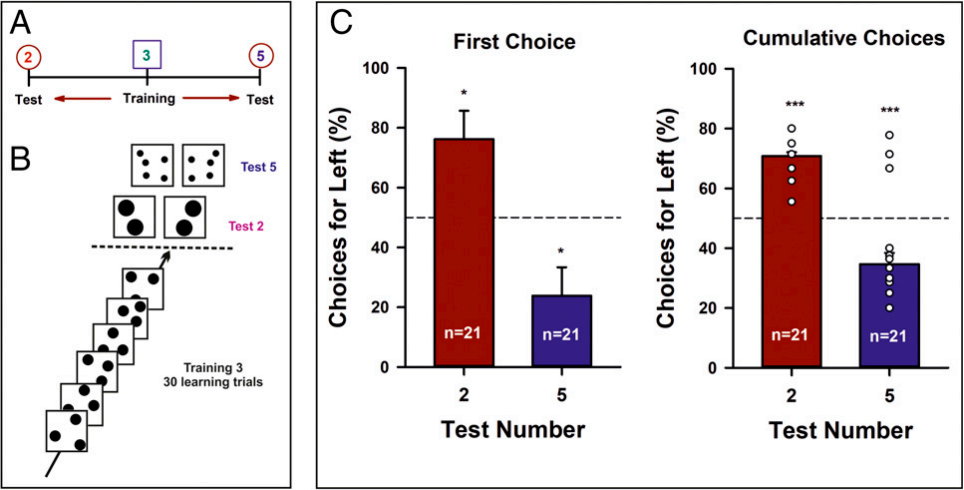
Fourth experiment. (*A*) Experimental protocol. Bees (*n* = 21) were trained on Three and then tested on Two or on Five using stimuli that were matched for nonnumeric cues such as perimeter, area, and density. (*B*) Experimental schedule (simplified). Bees were trained during 30 consecutive visits to the maze with a Three numerosity rewarded. Stimuli changed from visit to visit (e.g., spatial distribution, convex hull), but the Three numerosity remained constant and was associated with a sucrose reward. Once the training finished, bees were presented with two identical nonrewarded test stimuli. They were tested with a Two numerosity and with a Five numerosity in separate tests, with three refreshment trials interspersed between tests. Each test was repeated twice using different stimuli. (*C*) *Left*: Performance of bees when their first choice was recorded during a test opposing either Two vs. Two (red bar) or Five vs. Five (blue bar). The figure shows the percentage of the times a bee chose the *Left* panel. In the Two vs. Two test, bees preferred the *Left* panel. In the Five vs. Five test, bees preferred the *Right* panel. *Right*: Same as in the *Left* panel but for the cumulative choices recorded during 40 s in each test. *P < 0.03; ***P < 0.001.

To further control for multiple nonnumeric cues simultaneously (i.e., perimeter, area, and density), we performed a fourth experiment, which was similar to the first but with only circles used throughout and the single-item test stimuli of the first experiment replaced by two-item test stimuli to control for nonnumeric variables (Fig. 1*E* and *SI Appendix*, Fig. 4). Bees (*n* = 21) were trained on Three and then tested on Two and on Five (Fig. 5 *A* and *B*). In this case, each test was performed only once, using different shapes in either case (i.e., two tests per bee). Significant preference for Two on the left and for Five on the right would be consistent with a left-to-right number ordering. Bees exhibited a significant preference for Two on the left (Fig. 5*C*, Tables 1 and 2; training Three/test Two, first choice: empirical proportion of left choices, 0.76, *P* = 0.027, 95% CI, 0.52 to 0.92; cumulative choices: γ_1_ = −2.8, *P* < 0.001, *R*^2^c = 70.5%), whereas when the same bees were tested on Five, they preferred Five on the right (Fig. 5*C*, Tables 1 and 2; training Three/test Five, first choice: empirical proportion of right choices 0.76, *P* = 0.027, 95% CI, 0.52 to 0.92; cumulative choices: γ_1_ = 2.0, *P* < 0.001, *R*^2^c = 30.2%). This experiment further excluded the use of nonnumeric cues and confirmed that bees associated numeric magnitudes with space.

## Discussion

Investigations on the numeric sense of bees have revealed a rich spectrum of numeric competences (19, 21). For instance, bees relate large numbers (“more”) to large sizes and small numbers (“less”) to small sizes in tests in which the same number is presented but with different sizes (22), thus indicating that numbers and size are represented by a common magnitude system (23). We add here the existence of a left-to-right spatial representation of number, which provides further evidence for the convergence between the numeric sense of vertebrates and invertebrates. Bees may not count in the same way as vertebrates (24) (i.e., sequentially instead of “at a glance”), but they definitely can extract a number from visual targets.

Experiments on visual cognition with freely flying bees require training the bees to fly into setups to collect sucrose solution, as otherwise foragers would not come regularly to a place where food is not available. Training guarantees, in addition, that transfer tests with novel stimuli and in extinction conditions do not induce an immediate abandonment of the maze without completing the experiment. Novelty consisted here in displaying new, identical numerosities, which were spontaneously preferred by the bees on the left or on the right depending on their trained numerosity. This design, which has been used in other works on animal and human numerosity (4, 5), excludes criticisms on a lack of control of nonnumeric variables when animals decide between two test alternatives (25), given that the test stimuli were identical mirrored images. This was the case in three of our four experiments (Figs. 2, 3, and 5) where no difference in nonnumeric cues existed between test stimuli. In the remaining experiment (Fig. 4), test alternatives were different (a large vs. a small rectangle) yet conceived to control for nonnumeric cues such as area and perimeter. Moreover, in all experiments, control of these cues was achieved between training and tests (in particular in the fourth experiment), thus excluding a possible incidence of nonnumeric variables.

Brain hemispheric specialization in visual processing (26, 27) has been suggested as the cause of existence of an MNL (28). Lateralization at the level of visual-feature processing results in extraction of low spatial frequencies, typical of small quantities, with the right hemisphere (i.e., on the left visual field), while high spatial frequencies, typical of large quantities, are extracted with the left hemisphere (i.e., on the right visual field) (29–31). In honey bees, there is no interhemispheric chiasma; however, interhemispheric lateralization has also been reported in behavioral experiments, in particular for antennal olfactory processing (32) and gustatory processing (33), which are favored on the right antennal side. Accordingly, asymmetries in neural activity have been found in the olfactory pathways where projection-neuron activity differs between the left and right lateral antenno-cerebralis tracts so that a higher odorant discriminatory power is achieved on the right brain side (34). Lateralization in visual processing was reported for harnessed bees conditioned to associate a yellow rectangle with sucrose delivered to their proboscis. When one of the eyes of these bees was painted black, bees with the right eye free learned better the color association than bees with the left eye free, thus suggesting lateralization in favor of the right eye (35). Although it is still necessary to determine if this lateralization also applies to spatial frequencies, our results suggest that similar principles may exist in the bee brain in the case of visual information processing.

Overall, our results highlight the convergence of numeric processing strategies that exist across brains of different complexities despite evolutionary differences (36). Our results are consistent with findings reported in humans and vertebrates using a similar experimental design. For instance, 55-d-old babies familiarized with a 12 numerosity and presented with novel numerosities look preferentially at four on the left compared with four on the right, while they prefer to look at 36 on the right compared with 36 on the left. In doing so, they do not look at the side that would be closer to the familiar numerosity but rather express in their choice where, in an MNL, the novel numerosities would lie. The same response is exhibited by chicks familiarized with a five numerosity, which prefer two on the left in a two vs. two test and eight on the right in an eight vs. eight test. Like human babies, chicks do not choose based on the spatial vicinity to the familiar number but rather respond to novel numbers by assigning to them the spatial location defined by the MLN. Our results show that this strategy is common to bees and that the association between space and magnitude is consistent across various species, including humans, which argues in favor of a form of numeric representation rooted in the organization of lateralized nervous systems. A crucial issue is the origin of a left-to-right, rather than right-to-left, spatial numeric association in vertebrates (human and animals) and invertebrates (37). Although in principle arbitrary, the left-to-right mapping direction during evolution may have been imposed by brain asymmetry. Such a common and ancient trait, which occurs in a wide range of vertebrates and invertebrates, may have helped different species better process different kinds of information. We suggest, therefore, that the ability to order numbers spatially has probably emerged in different species exhibiting asymmetries in information processing between left and right brain hemispheres.

## Materials and Methods

### Animals.

Honey bee (*Apis mellifera*) foragers were trained to visit the experimental setup to collect sucrose solution in the middle of its backwall (Fig. 1*A*) in the absence of visual stimuli. Only highly motivated foragers returning regularly to the setup (typically every 3 to 5 min) were used to ensure efficient learning performances. Bees were individually marked with color spots on the thorax and/or abdomen. For every experiment, only one marked bee was allowed in the setup at a time.

### Apparatus.

The experiments were mostly done during the lockdown periods imposed by COVID-19. The setup was made of a wine box (Chateaux Fontarney; *SI Appendix*, Fig. 1*A*) that was transformed into a maze. It consisted of a wooden box ( × 24 × 20 cm) with a circular entrance (5 cm in diameter) in the middle of the front wall and a sliding door, which allowed excluding nondesired bees, other than the focal one (Fig. 1*A*). After entering the box, the bees accessed a first compartment with a similar entrance on its back wall. Through this second entrance, the trained bee accessed a second compartment (the decision chamber) in which the visual stimuli were presented. The distance between the stimuli and the second entrance was 15 cm, so that each visual image subtended a visual angle of 30° to the eye of a bee located at the entrance of the decision chamber. The box was covered with a glass ceiling ensuring daylight illumination within the maze. Sucrose solution (50% weight per weight) was delivered in the center of the back wall of the decision chamber by means of an Eppendorf tip, 5 mm in diameter. Two diagonal panels in the decision chamber allowed us to present simultaneously two visual targets during a test (Fig. 1*B*). Both during the training and during the tests, the experimenter always stood behind the maze to leave a free way to the entrance and was aligned with the maze’s main axis to avoid inducing left/right biases.

### Stimuli.

Stimuli were black and white patterns made of circles, squares, and triangles presented on a 8 × 8 cm white square-shaped background, which subtended a visual angle of 30° to the eyes of a bee entering the last maze compartment (Figs. 1 *C* and *D*). When items of different sizes were presented on a same image (first experiment, Fig. 1C; 2.5, 2, and 1.5 cm for largest extent), they subtended visual angles of 9.5°, 7.6°, and 5.7°, respectively, which rendered them well resolvable (38). The same stimuli were used in the first and second experiments (Fig. 1*C* and *SI Appendix*, Fig. 2). They were matched in terms of total area (1,250 mm^2^), which was the same in all training and test stimuli presenting different numbers. They were varied in terms of convex hull to avoid the use of this cue. They differed in total perimeter (squares: 240 mm; circles: 212.7 mm; triangles: 290 mm), so that in a third experiment, targets were designed to control for perimeter and area. In this case, training stimuli displayed either three squares or three triangles with different spatial arrays (Fig. 1*D*, Training; *SI Appendix*, Fig. 3). Each item had a perimeter of 8 cm (i.e., a total perimeter of 24 cm per image). Test stimuli were two horizontal rectangles, one with a perimeter of 12 cm and the other with a perimeter of 24 cm (Fig. 1*D*, Test). The area of the rectangles also differed as it was twice as large in the larger rectangle. In the fourth experiment, the stimuli were created in Arc Gis 10.6.1 (*SI Appendix*, Fig. 4). Training stimuli consisted of three circles, each 26.67 mm in diameter, while test stimuli displayed either two circles, each 40 mm in diameter, or five circles, each 16 mm in diameter. Thus, the summed perimeter of all circles was identical in each stimulus, 251.3 mm, while the total area of the dots inversely correlated with number: two circles = 251.3 mm^2^, three circles =167.6 mm^2^, and five circles = 100.5 mm^2^. The mean distance between the edge of each dot and the nearest edge of each other dot was identical (22.93 mm) in each stimulus so that density was equal across training and test stimuli.

### Training Phase.

A single image was presented in the middle of the back wall of the decision chamber. The image provided a sucrose reward in the middle (*SI Appendix*, Fig. 1*C*). The reference number presented during the training trials remained constant, but the stimuli used to display it varied from trial to trial (e.g., circles, squares, or triangles in the first and second experiment, or triangles and squares in the third experiment) to force the bees to focus on number, irrespective of pattern features. Training consisted of 30 consecutive rewards (i.e., 30 consecutive visits to the setup) delivered on the reference number shown on the back wall of the decision chamber. After each training trial, the bee left the setup, and the training stimulus was replaced by a new, different one, displaying the same number of items.

### Test Phase.

The test phase consisted of nonrewarded presentations of two stimuli displayed on the diagonal, lateral panels of the decision chamber (Fig. 1*B*). Three refreshment trials were performed between tests to keep the bee motivated to visit the setup. During each refreshment trial, the reference number was again presented and the bee was rewarded for landing on it. In this way, its foraging motivation was maintained despite the negative experience of a nonrewarded test. Fresh stimuli were used for every test to avoid the use of scent marks. In all experiments except the third, test alternatives on the left and the right panels were identical mirrored images, which varied between tests. During each test, we recorded the first choice performed by the focal bee as well as the cumulative choices of each panel during 40 s. This period of time is used commonly in visual tests performed with free-flying bees and is kept intentionally short as bees may change their search strategy after repeated choices followed by the absence of reward (16). Choices were the contacts made by the bee with the surface of the targets.

### Statistical Analysis.

With the exception of experiment 4, in all experiments, each test (e.g., One vs. One or Five vs. Five) was repeated twice. Thus, those data could be considered as not independent (i.e., the values on level 1 [repeated measures] are nested in a bee [level 2]). In order to test for independence of the first-choice data, we computed intraclass correlation coefficients (ICCs) for count data in generalized linear mixed models across the experiments. The ICC showed that the between-subjects variance (level 2) was extremely low (the range across all experiments was between 1.8 × 10^−9^ and 5.2 × 10^−10^), indicating independence in the data. Therefore, first-choice data were analyzed with a two-sided exact binomial test (including experiment 4) that compared empirical test proportions with an expected proportion of 0.5 (i.e., assuming a random choice between left and right).

The ICC was also computed for the cumulative-choice data (except for experiment 4). Between-subjects variance ranged between 0% and 4%. As 1% variance on level 2 is generally seen as a threshold for data interdependence (37), we used a multilevel modeling approach (38) to analyze whether bees chose more often the left or right test stimuli. The equation used for these multilevel models was as follows:

$$\mathrm{Cumulative}\;\mathrm{choice}s_{\text{it}}=\gamma_{0}+\gamma_{1}\text{left}-{\text{right}}_{\text{it}}+{\text{u}}_{0\text{i}}+\varepsilon_{\text{it}},$$

where *i* corresponds to individual bees and *t* to the time (i.e., repeated measures), γ_0_ represents the intercept, γ_1_ represents the effect “flying to the left or to the right,” u_0i_ represents the random intercept, and *ε*_it_ the regression residual for each bee *i* at time *t*. A simple regression model was used for experiment 4.

We used R, version 4.1.2 (39), the *psych* package for descriptive statistics (40), and the packages *lme4* (41) and *lmerTest* (42) for multilevel modeling. Although all the hypotheses were directional, all results were tested conservatively using two-tailed hypotheses.

## Supplementary Material

Supplementary File

## Data Availability

The data sets as well as the R scripts [Test Performances (First Choices and Cumulative Choices) R syntax scripts] generated during this study are available at the following FIGSHARE repository link: https://doi.org/10.6084/m9.figshare.19249133 (43).
