# Supplementary material for: An insect brain organizes numbers on a left-to-right mental number line
Source: Proc Natl Acad Sci U S A. 2022 Oct 17;119(44):e2203584119. doi: 10.1073/pnas.2203584119 (PMC9636979; doi:10.1073/pnas.2203584119)
Supplement: Supplementary File [file pnas.2203584119.sapp.pdf]

PNAS
www.pnas.org

An insect brain organizes numbers on a left-to-right mental number line

Martin Giurfa[1,2], Claire Marcout[1], Peter Hilpert[3], Catherine Thevenot[3]* and Rosa Rugani[4]*

[1] *Centre de Recherches sur la Cognition Animale, Centre de Biologie Intégrative (CBI), University of Toulouse, CNRS, UPS, 31062 Toulouse cedex 9, France.*

[2] *Institut Universitaire de France (IUF), Paris, France.*

[3] *Institute of Psychology, University of Lausanne, CH-1015, Lausanne, Switzerland.*

[4] *Department of General Psychology, University of Padova, 35100 Padova, Italy*

*Shared senior authorship

Corresponding author: Martin GIURFA

Email: martin.giurfa@univ-tlse3.fr

Supplementary Materials include :

- Supplementary Figure 1
- Supplementary Figure 2
- Supplementary Figure 3
- Supplementary Figure 4

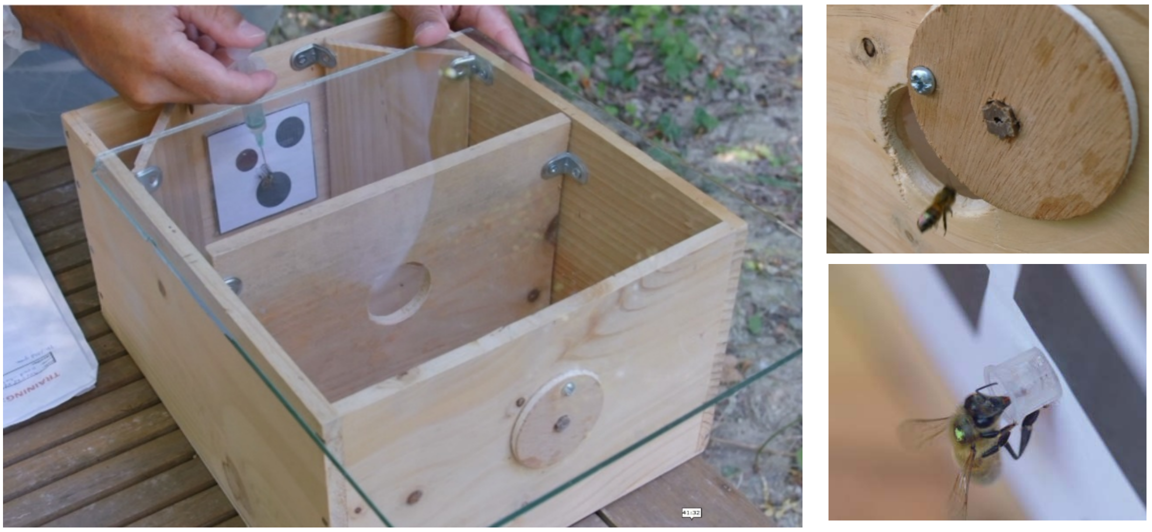

Supplementary Fig. 1. A) View of the maze and positioning of the experimenter during behavioral recordings. The experimenter stood always behind the maze, to leave a free-way to the entrance, and was aligned with the maze’s main axis, to avoid inducing left/right biases. **B)** Trained bee entering the maze. **C)** Trained bee rewarded on a square stimulus.

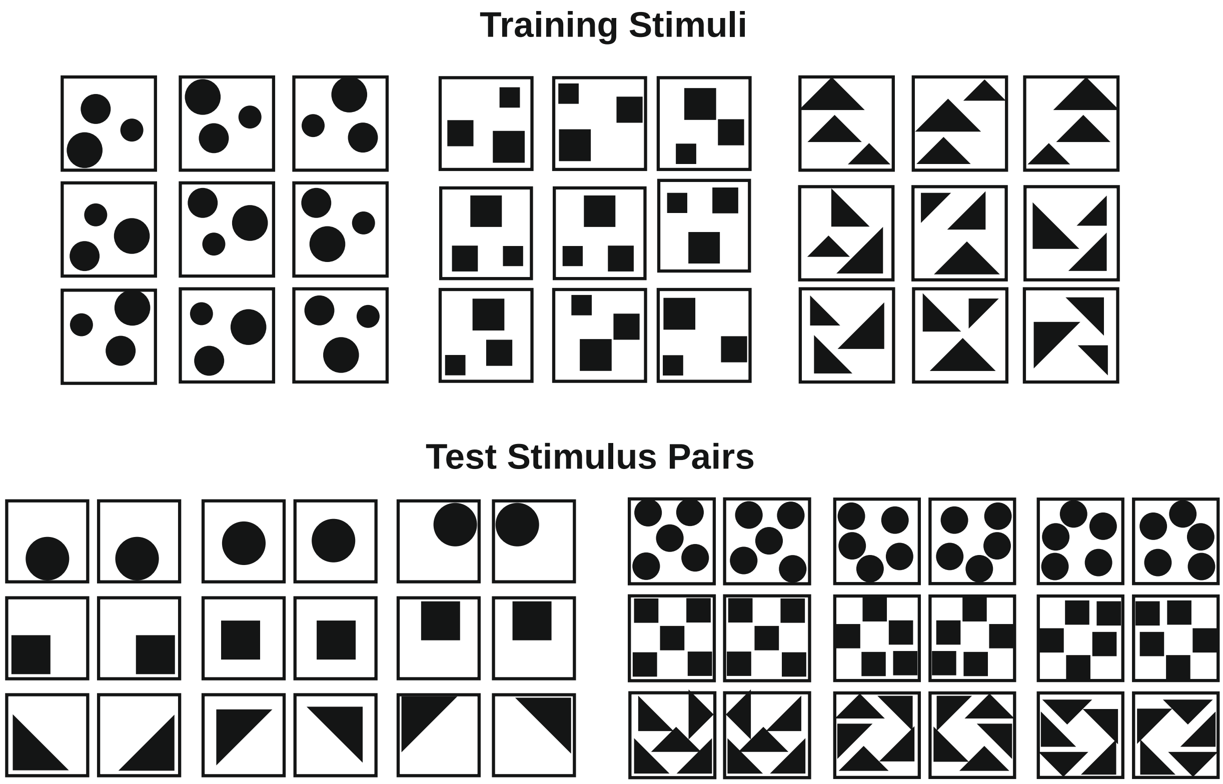

Supplementary Fig. 2. Training and test stimuli used in the **first experiment** (Fig. 2, main text) in which bees were trained on Three and tested on One and on Five. In the **second experiment** (Fig. 3, main text), in which one group of bees was trained on One and tested on Three, the One stimuli shown in the lower three rows (left three columns) were used for training. In the group trained on Five and tested on Three, the Five stimuli shown in the lower three rows (right three columns) were used for training. Both groups were tested on Three using the Three stimuli of the upper three rows presented as identical mirrored images.

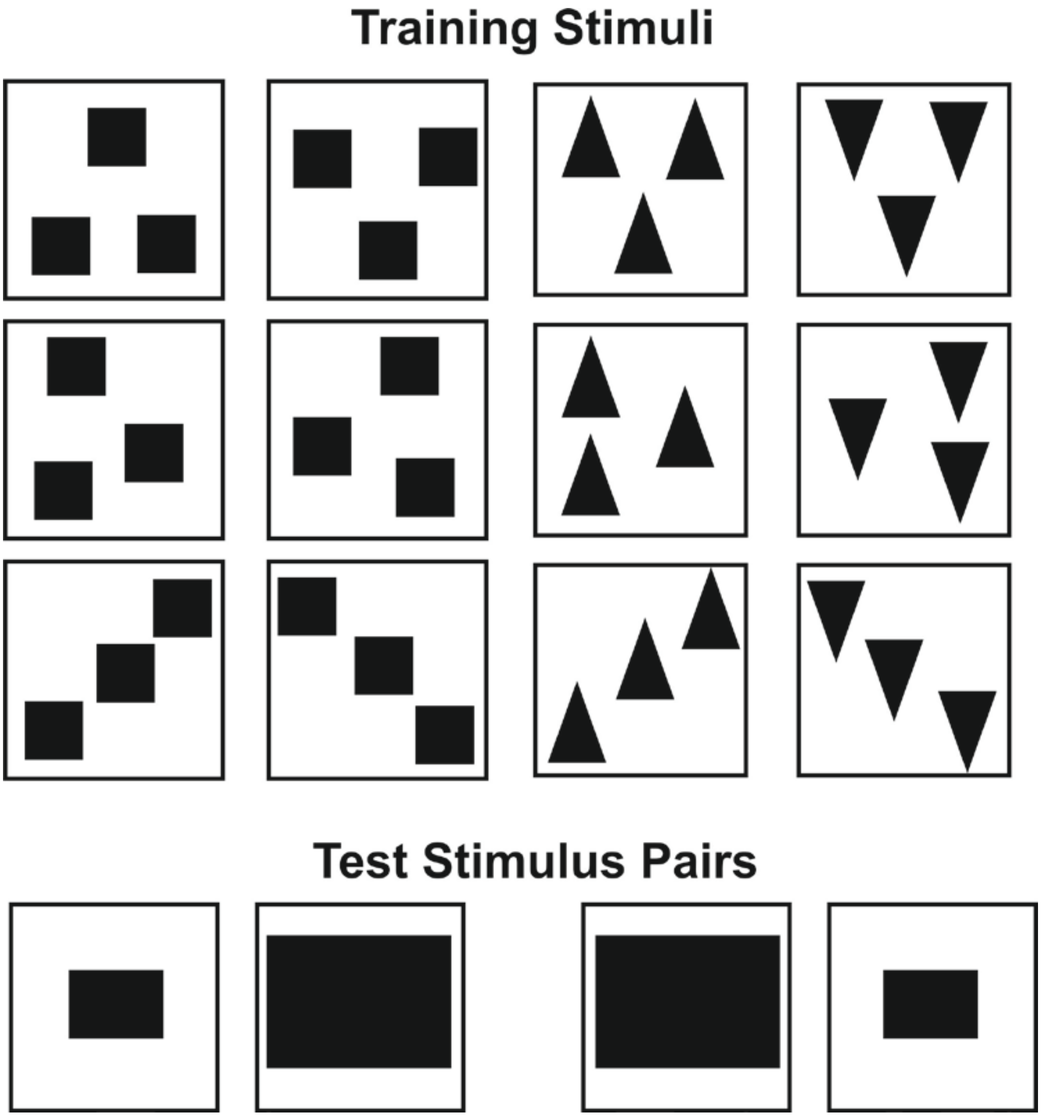

Supplementary Fig. 3. Training and test stimuli used in the third experiment (Fig. 4, main text).

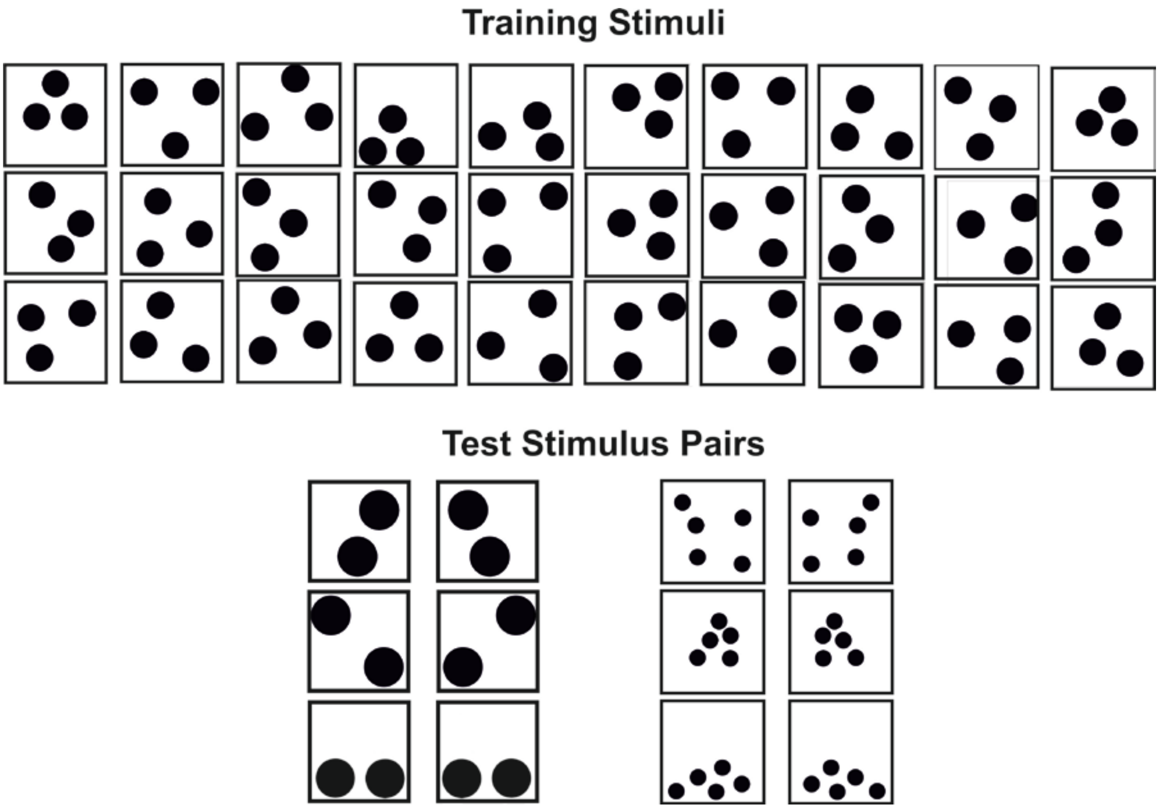

Supplementary Fig. 4. Training and test stimuli used in the fourth experiment (Fig. 5, main text).
